# Guanidinium 4-amino­benzoate

**DOI:** 10.1107/S160053681000396X

**Published:** 2010-02-06

**Authors:** P. S. Pereira Silva, M. Ramos Silva, J. A. Paixão, A. Matos Beja

**Affiliations:** a CEMDRX, Physics Department, University of Coimbra, P-3004-516 Coimbra, Portugal

## Abstract

In the title compound, CH_6_N_3_^+^·C_7_H_6_NO_2_^−^, the cation and anion lie on crystallographic mirror planes. The 4-amino­benzoate anion is almost in a planar conformation with a maximum deviation of 0.024 (2) Å for the N atom. The bond length in the deprotonated carboxyl group is inter­mediate between those of normal single and double Csp^2^=O bonds, indicating delocalization of the charge over both O atoms of the COO^−^ group. In the crystal, N—H⋯O hydrogen bonds assemble the ions in layers propagating in the *bc* plane. This structure is very similar to that of guanidinium benzoate.

## Related literature

For a related structure, see: Pereira Silva *et al.* (2007 ▶). 4-Amino­benzoic acid has two known polymorphs, see: Gracin & Rasmuson (2004 ▶). For the potential applications of guanidine compounds in non-linear optics, see: Zyss *et al.* (1993 ▶). For bond-length data, see: Allen *et al.* (1987 ▶).
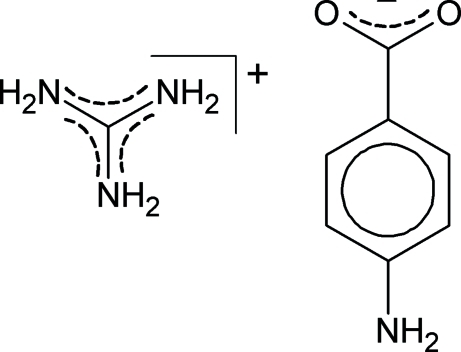


## Experimental

### 

#### Crystal data

CH_6_N_3_^+^·C_7_H_6_NO_2_^−^
*M* *_r_* = 196.22
Orthorhombic, 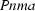

*a* = 14.9833 (4) Å
*b* = 8.0602 (2) Å
*c* = 8.4737 (2) Å
*V* = 1023.36 (4) Å^3^
*Z* = 4
Mo *K*α radiation
μ = 0.10 mm^−1^
*T* = 293 K
0.33 × 0.19 × 0.15 mm

#### Data collection

Bruker APEXII CCD area-detector diffractometer
Absorption correction: multi-scan (*SADABS*; Sheldrick, 2003 ▶) *T*_min_ = 0.898, *T*_max_ = 0.986
19879 measured reflections
1323 independent reflections
960 reflections with *I* > 2σ(*I*)
*R*_int_ = 0.026

#### Refinement

*R*[*F* ^2^ > 2σ(*F* ^2^)] = 0.037
*wR*(*F* ^2^) = 0.109
*S* = 1.04
1323 reflections
85 parameters
H atoms treated by a mixture of independent and constrained refinement
Δρ_max_ = 0.13 e Å^−3^
Δρ_min_ = −0.20 e Å^−3^

### 

Data collection: *APEX2* (Bruker, 2003 ▶); cell refinement: *SAINT* (Bruker, 2003 ▶); data reduction: *SAINT*; program(s) used to solve structure: *SHELXS97* (Sheldrick, 2008 ▶); program(s) used to refine structure: *SHELXL97* (Sheldrick, 2008 ▶); molecular graphics: *PLATON* (Spek, 2009 ▶); software used to prepare material for publication: *SHELXL97*.

## Supplementary Material

Crystal structure: contains datablocks global, I. DOI: 10.1107/S160053681000396X/ds2019sup1.cif

Structure factors: contains datablocks I. DOI: 10.1107/S160053681000396X/ds2019Isup2.hkl

Additional supplementary materials:  crystallographic information; 3D view; checkCIF report

## Figures and Tables

**Table 1:** Hydrogen-bond geometry (Å, °)

*D*—H⋯*A*	*D*—H	H⋯*A*	*D*⋯*A*	*D*—H⋯*A*
N1—H1*A*⋯O1^i^	0.940 (15)	1.869 (16)	2.8068 (14)	175.4 (13)
N1—H1*B*⋯O1^ii^	0.875 (16)	2.107 (16)	2.9032 (15)	151.0 (13)
N2—H2*A*⋯O1^ii^	0.923 (16)	2.099 (16)	2.9408 (8)	151.1 (12)

Symmetry codes: (i) 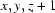; (ii) 
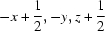
.

**Fig. 1.**
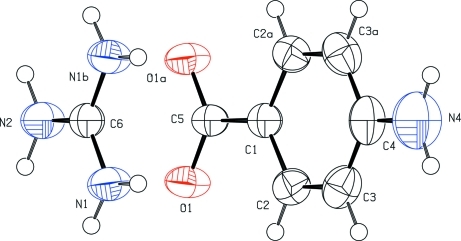
A plot of the title compound. Displacement ellipsoids are drawn at the 50% probability level. [Symmetry codes: (a);(b) x, 1/2-y, z]

**Fig. 2.**
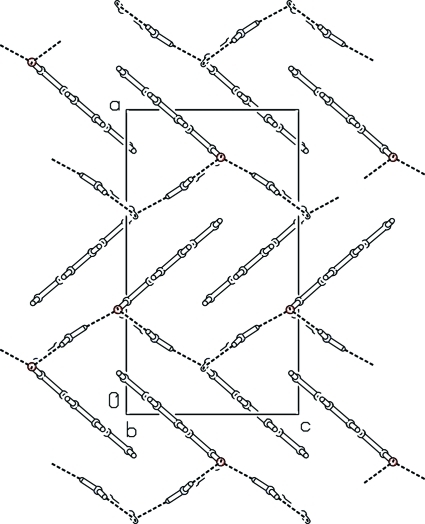
A packing diagram for (I), viewed down the b axis, showing the layer formation. Hydrogen bonds are shown as dashed lines.

**Fig. 3.**
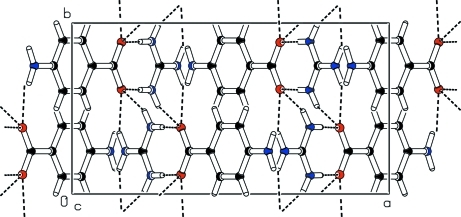
A packing diagram for (I), viewed down the c axis, with the hydrogen bonds depicted by dashed lines.

## supplementary crystallographic information

### Comment

Guanidine is a strong Lewis base and the guanidinium cation may be easly anchored onto numerous inorganic and organic anions and polyanions, largely because of the presence of six potential donor sites for hydrogen-bonding interactions. From the point of view of their physical properties, guanidine compounds are potentially interesting for non-linear optics applications since guanidinium, a polarizable acentric two-dimensional cation, can be regarded as a planar octupolar chemical entity (Zyss *et al.*, 1993). We are currently engaged in a research project aimed at investigating the structures, and the dielectric and optical properties of guanidine and guanidine derivative compounds.

4-Aminobenzoic acid has two known polymorphs (Gracin & Rasmuson, 2004) and is one of the most versatile acids for structure extension by linear hydrogen-bonding associations, through both the carboxylic acid and the amine functional groups.

Both ions of the title compound, (I), Fig. 1, possess mirror symmetry, with the C6 and N2 atoms of the cation situated in the mirror plane as well as the carboxylate group C atom, the ipso-C and the para-C and N atoms of the anion.

The 4-aminobenzoate anion is almost in a planar conformation, with the atoms N4 and C5 displaced from the ring plane by about the same amounts, 0.024 (2) and 0.022 (2)Å respectively, and in the same direction.

The dihedral angle between the phenyl ring and the carboxylate group [1.58 (16)°] is slightly larger than the corresponding angle in guanidinium benzoate [0.41 (18)°].

The O—C—O angle of the carboxylate group is greater than 120° because of the steric effect of lone-pair electrons on both O atoms. The bond length in the deprotonated carboxyl group is intermediate between the single Csp^2^—O (1.308-1.320 Å) and double Csp^2^═O bond lengths (1.214-1.224 Å, Allen *et al.*, 1987), indicating delocalization of the charge over both O atoms of the COO^-^ group.

The three C—N bond lengths in the propeller-shaped (CH_6_N_3_)^+^ cation are similar (Table 1), the symmetry of the cation being C_3 h_. The usual model of electron delocalization in this species, leading to a C—N bond order of 1.33, is applicable here.

All H atoms on the guanidinium cation are involved in N—H···O interactions with the anion (Fig. 2, Table 2) forming infinite layers propagating in the bc plane, each carboxylate O atom accepting three hydrogens. In each layer the cation is bonded to three anions, two approximately perpendicular and one approximately coplanar (Fig. 3). This pattern is also found in guanidinium benzoate (Pereira Silva *et al.*, 2007).

### Experimental

The title compound was prepared by adding 4-aminobenzoic acid (Aldrich, 99%, 1.0 mmol) to guanidinium carbonate (Aldrich 99%, 0.5 mmol) in a water solution (100 ml). The solution was slowly warmed to the boiling point and then left to evaporate under ambient conditions. After 2 weeks, small light brown single crystals were obtained.

### Refinement

All H atoms were located in a difference Fourier synthesis. The guanidinium H-atom coordinates were refined, with U_iso_(H) = 1.2U_eq_(N). The H atoms of the anion were placed in calculated positions and refined as riding on their parent atoms, using SHELXL97 (Sheldrick, 2008) defaults [C—H = 0.93 Å and U_iso_(H) = 1.2U_eq_(C)].

### Crystal data

**Table:**

CH_6_N_3_^+^·C_7_H_6_NO_2_^−^	*F*(000) = 416
*M**_r_* = 196.22	*D*_x_ = 1.274 Mg m^−^^3^
Orthorhombic, *P**n**m**a*	Mo *K*α radiation, λ = 0.71073 Å
Hall symbol: -P 2ac 2n	Cell parameters from 8009 reflections
*a* = 14.9833 (4) Å	θ = 2.8–27.5°
*b* = 8.0602 (2) Å	µ = 0.10 mm^−^^1^
*c* = 8.4737 (2) Å	*T* = 293 K
*V* = 1023.36 (4) Å^3^	Irregular edge, light brown
*Z* = 4	0.33 × 0.19 × 0.15 mm

### Data collection

**Table:**

Bruker APEX2 CCD area-detector diffractometer	1323 independent reflections
Radiation source: fine-focus sealed tube	960 reflections with *I* > 2σ(*I*)
graphite	*R*_int_ = 0.026
φ and ω scans	θ_max_ = 28.1°, θ_min_ = 2.7°
Absorption correction: multi-scan (*SADABS*; Sheldrick, 2003)	*h* = −18→19
*T*_min_ = 0.898, *T*_max_ = 0.986	*k* = −10→9
19879 measured reflections	*l* = −10→11

### Refinement

**Table:**

Refinement on *F*^2^	Primary atom site location: structure-invariant direct methods
Least-squares matrix: full	Secondary atom site location: difference Fourier map
*R*[*F*^2^ > 2σ(*F*^2^)] = 0.037	Hydrogen site location: inferred from neighbouring sites
*wR*(*F*^2^) = 0.109	H atoms treated by a mixture of independent and constrained refinement
*S* = 1.04	*w* = 1/[σ^2^(*F*_o_^2^) + (0.051*P*)^2^ + 0.1635*P*] where *P* = (*F*_o_^2^ + 2*F*_c_^2^)/3
1323 reflections	(Δ/σ)_max_ < 0.001
85 parameters	Δρ_max_ = 0.13 e Å^−^^3^
0 restraints	Δρ_min_ = −0.20 e Å^−^^3^

### Special details

**Table:**

Geometry. All esds (except the esd in the dihedral angle between two l.s. planes) are estimated using the full covariance matrix. The cell esds are taken into account individually in the estimation of esds in distances, angles and torsion angles; correlations between esds in cell parameters are only used when they are defined by crystal symmetry. An approximate (isotropic) treatment of cell esds is used for estimating esds involving l.s. planes.
Refinement. Refinement of *F*^2^ against ALL reflections. The weighted *R*-factor wR and goodness of fit *S* are based on *F*^2^, conventional *R*-factors *R* are based on *F*, with *F* set to zero for negative *F*^2^. The threshold expression of *F*^2^ > σ(*F*^2^) is used only for calculating *R*-factors(gt) etc. and is not relevant to the choice of reflections for refinement. *R*-factors based on *F*^2^ are statistically about twice as large as those based on *F*, and *R*- factors based on ALL data will be even larger.

### Fractional atomic coordinates and isotropic or equivalent isotropic displacement parameters (Å^2^)

**Table:**

	*x*	*y*	*z*	*U*_iso_*/*U*_eq_	
O1	0.34385 (6)	0.11290 (10)	−0.04842 (10)	0.0519 (3)	
C1	0.43652 (10)	0.2500	0.13590 (17)	0.0390 (4)	
C2	0.46854 (8)	0.10235 (14)	0.19926 (13)	0.0462 (3)	
H2	0.4490	0.0019	0.1577	0.055*	
C3	0.52869 (8)	0.10183 (16)	0.32253 (14)	0.0542 (3)	
H3	0.5491	0.0015	0.3630	0.065*	
C4	0.55904 (11)	0.2500	0.3867 (2)	0.0556 (5)	
C5	0.37086 (10)	0.2500	0.00390 (17)	0.0391 (4)	
N4	0.61881 (15)	0.2500	0.5120 (3)	0.0886 (7)	
H4	0.6353 (13)	0.149 (3)	0.543 (2)	0.106*	
N1	0.25025 (7)	0.10833 (13)	0.66469 (14)	0.0551 (3)	
H1A	0.2845 (9)	0.1093 (16)	0.7576 (18)	0.066*	
H1B	0.2285 (9)	0.016 (2)	0.6258 (17)	0.066*	
N2	0.17018 (11)	0.2500	0.47745 (19)	0.0563 (4)	
H2A	0.1493 (9)	0.147 (2)	0.4470 (16)	0.068*	
C6	0.22330 (11)	0.2500	0.6031 (2)	0.0426 (4)	

### Atomic displacement parameters (Å^2^)

**Table:**

	*U*^11^	*U*^22^	*U*^33^	*U*^12^	*U*^13^	*U*^23^
O1	0.0705 (6)	0.0334 (4)	0.0519 (5)	−0.0031 (4)	−0.0139 (4)	−0.0006 (3)
C1	0.0415 (8)	0.0398 (8)	0.0358 (7)	0.000	0.0050 (6)	0.000
C2	0.0489 (6)	0.0455 (6)	0.0442 (6)	0.0021 (5)	0.0014 (5)	0.0013 (5)
C3	0.0530 (7)	0.0627 (8)	0.0467 (7)	0.0100 (6)	−0.0004 (5)	0.0089 (6)
C4	0.0443 (9)	0.0813 (14)	0.0411 (9)	0.000	−0.0018 (8)	0.000
C5	0.0460 (8)	0.0335 (8)	0.0380 (8)	0.000	0.0035 (7)	0.000
N4	0.0818 (13)	0.1110 (19)	0.0732 (13)	0.000	−0.0344 (11)	0.000
N1	0.0655 (7)	0.0379 (6)	0.0618 (7)	0.0026 (5)	−0.0206 (5)	−0.0012 (5)
N2	0.0656 (10)	0.0446 (9)	0.0587 (9)	0.000	−0.0226 (8)	0.000
C6	0.0419 (8)	0.0398 (8)	0.0460 (8)	0.000	−0.0036 (7)	0.000

### Geometric parameters (Å, °)

**Table:**

O1—C5	1.2575 (11)	C4—N4	1.390 (3)
C1—C2^i^	1.3910 (13)	C5—O1^i^	1.2575 (11)
C1—C2	1.3910 (13)	N4—H4	0.89 (2)
C1—C5	1.490 (2)	N1—C6	1.3188 (13)
C2—C3	1.3796 (17)	N1—H1A	0.940 (15)
C2—H2	0.9300	N1—H1B	0.875 (16)
C3—C4	1.3886 (15)	N2—C6	1.329 (2)
C3—H3	0.9300	N2—H2A	0.923 (16)
C4—C3^i^	1.3886 (15)	C6—N1^i^	1.3188 (13)
			
C2^i^—C1—C2	117.65 (14)	O1—C5—O1^i^	122.98 (14)
C2^i^—C1—C5	121.18 (7)	O1—C5—C1	118.50 (7)
C2—C1—C5	121.18 (7)	O1^i^—C5—C1	118.50 (7)
C3—C2—C1	121.34 (11)	C4—N4—H4	113.7 (13)
C3—C2—H2	119.3	C6—N1—H1A	119.5 (8)
C1—C2—H2	119.3	C6—N1—H1B	118.1 (10)
C2—C3—C4	120.50 (12)	H1A—N1—H1B	121.7 (13)
C2—C3—H3	119.7	C6—N2—H2A	115.3 (9)
C4—C3—H3	119.7	N1^i^—C6—N1	119.95 (15)
C3—C4—C3^i^	118.64 (15)	N1^i^—C6—N2	120.02 (8)
C3—C4—N4	120.68 (8)	N1—C6—N2	120.02 (8)
C3^i^—C4—N4	120.68 (8)		
			
C2^i^—C1—C2—C3	1.2 (2)	C2^i^—C1—C5—O1	−179.70 (13)
C5—C1—C2—C3	−179.36 (12)	C2—C1—C5—O1	0.8 (2)
C1—C2—C3—C4	−0.1 (2)	C2^i^—C1—C5—O1^i^	−0.8 (2)
C2—C3—C4—C3^i^	−1.0 (3)	C2—C1—C5—O1^i^	179.70 (13)
C2—C3—C4—N4	179.23 (16)		

Symmetry codes: (i) *x*, −*y*+1/2, *z*.

### Hydrogen-bond geometry (Å, °)

**Table:**

*D*—H···*A*	*D*—H	H···*A*	*D*···*A*	*D*—H···*A*
N1—H1A···O1^ii^	0.940 (15)	1.869 (16)	2.8068 (14)	175.4 (13)
N1—H1B···O1^iii^	0.875 (16)	2.107 (16)	2.9032 (15)	151.0 (13)
N2—H2A···O1^iii^	0.923 (16)	2.099 (16)	2.9408 (8)	151.1 (12)

Symmetry codes: (ii) *x*, *y*, *z*+1; (iii) −*x*+1/2, −*y*, *z*+1/2.
